# Evaluation of Rapid Diagnostic Tests for Assessment of Hepatitis B in Resource-Limited Settings

**DOI:** 10.5334/aogh.2562

**Published:** 2019-07-04

**Authors:** James S. Leathers, M. Belen Pisano, Viviana Re, Gertine van Oord, Amir Sultan, Andre Boonstra, Jose D. Debes

**Affiliations:** 1 Department of Emergency Medicine, UC Davis School of Medicine, UC Davis Health, Sacramento, CA, US; 2 Virology Institute “Dr. J.M. Vanella”, Faculty of Clinical Sciences, CONICET, Universidad Nacional de Córdoba, Córdoba, AR; 3 Department of Gastroenterology and Hepatology, Erasmus MC, Rotterdam, NL; 4 Department of Gastroenterology, Addis Ababa University, Addis Ababa, ET; 5 Department of Medicine, University of Minnesota, Minneapolis, MN, US

## Abstract

Chronic Hepatitis B (HBV) is the most important cause of liver disease worldwide. There is a need for low-cost tests to aid in diagnosis and management of HBV infection in resource-limited settings. We evaluated the utility of several rapid diagnostic tests (RDT) in three different continents (Europe, South America, Africa). The HBsAg RDT showed optimal sensitivity and specificity. The anti-HBeAb RDT showed acceptable sensitivity and excellent specificity. Our results suggest that these RDTs could be used for screening and management of HBV.

Dear Editor,

Chronic Hepatitis B virus (HBV) infection is the most frequent cause of liver disease and hepatocellular carcinoma (HCC) worldwide, with most cases occurring in resource-limited settings [3]. Early diagnosis is critical in reducing hepatitis-related morbidity and mortality. Ironically, regions of the world with the largest HBV burden (i.e. Africa) are the regions with suboptimal laboratory infrastructure, leading to lack of diagnosis [4]. Past studies of HBV rapid tests have shown highly variable results and elevated costs for these tests make them often unaffordable in resource-limited regions [1,2]. We evaluated the efficacy of low-cost rapid diagnostic tests (RDTs) for HBV in Europe, Africa, and South America.

We performed an external validation of RDTs designed for the detection of various HBV serological markers (PRECHEK Bio. Inc., Korea). These RDTs were selected because of their low cost (approximately 7–28% of the cost of WHO-recommended RDTs) and their ability to be used for point-of-care diagnostics. These RDTs are immunochromatographic assays in which monoclonal antibodies against specific antigens or antibodies are immobilized on the test line of a nitrocellulose membrane pad. In positive tests, as serum/blood is added, the antigen-antibody complex migrates towards the test zone (T) where it is captured by immobilized antibodies, forming a visible line. In negative tests, the antigen or antibody is absent and there is no visible line.

HBV serological markers tested included HBV-surface antigen (HBsAg) HBV-surface antigen antibody (anti-HBsAb), HBV E antigen (HBeAg) and HBV E antibody (anti-HBeAb). Serum and whole-blood samples used for testing were obtained from repositories (stored at –80C) in hospitals in the Netherlands, Argentina, and Ethiopia. Testing was discontinued in RDTs that performed poorly during initial assessment. The performance of RDTs was assessed by ROC curve analysis, using the local diagnostic standard as the reference test (Argentina: ARCHITECT Reagent kits [Abbott, Germany]; Netherlands: LIAISON XL system [Diasorin, Italy]; Ethiopia: Onsite Rapid Test [CTK Biotech, USA]). Statistical analyses were performed using STATA v15.1 (Statacorp, College Station, TX).

A total of 200 unique serum and whole-blood samples were tested using RDTs. The median age of patients was 40 years (IQR 31–50) and 67% were male. HBV genotypes A-F were tested. The HBsAg serum strip had a sensitivity and specificity of 100%. The median HBsAg level of tested samples (in those available) was 2800 IU/mL (range: 150–110,000). The anti-HBeAb serum cassette had a sensitivity of 80% and a specificity of 100%. The HBsAg whole-blood cassette and strip had specificities of 100%, but sensitivities of 56% and 45%, respectively. The anti-HBsAb serum cassette had a sensitivity of 57% and a specificity of 93%. The anti-HBsAb serum strip had a sensitivity of 20% and a specificity of 100%. The HBeAg serum strip had a sensitivity of 81% and a specificity of 67%. The median HBeAg level of tested samples (in those available) was 2806 IU/mL (range: 1952–3149). Specific RDT performance is available in Table 1.

**Table 1:** Rapid Diagnostic Test Performance.

Test Type (Catalog Number)	Number^1^ Tested (T/P/N)	Test Site^2^(A/E/N)	Age^3^	Male	Sensitivity	Specificity

**HBsAg** *Serum Strip* *(HBV 211)*	81/55/26	A/E/N	39	74%	100%	100%
**HBsAg** *WB Cass*. *(HBV 214)*	23/16/7	A/N	43	70%	56%	100%
**HBsAg** *WB Strip* *(HBV 213)*	13/11/2	A/N	42	54%	45%	100%
**Anti-HBsAb** *Serum Cass*. *(HBV 222)*	38/23/15	N	52	58%	57%	93%
**Anti-HBsAb** *Serum Strip* *(HBV 221)*	46/20/26	N	38	80%	20%	100%
**Anti-HBeAb** *Serum Cass*. *(HBV 232)*	64/20/44	N	37	63%	80%	100%
**HBeAg** *Serum Strip* *(HBV 242)*	27/16/11	A/N	39	81%	82%	67%

^1^ T = total, P = known positive, N = known negative; ^2^ A = Argentina, E = Ethiopia, N = Netherlands; ^3^ Median age. HBsAg, hepatitis B surface antigen; anti-HBsAb, hepatitis B surface antibody; HBeAg, hepatitis B e antigen; Cass., cassette; WB, whole-blood.

The HBsAg serum strip RDT demonstrated optimal sensitivity and specificity in the three different continents, indicating that it can reliably diagnose HBV in various populations with different genotypes. The anti-HBeAb RDT showed acceptable sensitivity and excellent specificity, making it useful to differentiate HBeAb status. Overall, whole-blood HBsAg and serum anti-HBsAb kits performed poorly, as they were specific but insufficiently sensitive to be clinically useful for screening. The serum HBeAg kits demonstrated acceptable sensitivity, but poor specificity, making them unlikely to be useful in the clinical setting. Our results suggest that HBsAg and anti-HBeAb serum RDTS are reliable and, in conjunction with alanine aminotransferase levels (ALTs), can be useful for diagnosis, as well as informing the need for treatment in resource-limited settings.

## Data Accessibility Statements

Study data will be made available upon request of the corresponding author.
